# Fever: Views in Anthroposophic Medicine and Their Scientific Validity

**DOI:** 10.1155/2016/3642659

**Published:** 2016-11-24

**Authors:** David D. Martin

**Affiliations:** ^1^University Children's Hospital, Tübingen, Germany; ^2^Filderklinik, Filderstadt, Germany

## Abstract

*Objective*. To conduct a scoping review to characterize how fever is viewed in anthroposophic medicine (AM) and discuss the scientific validity of these views.* Methods*. Systematic searches were run in Medline, Embase, CAMbase, and Google Scholar. Material from anthroposophic medical textbooks and articles was also used. Data was extracted and interpreted.* Results*. Most of the anthroposophic literature on this subject is in the German language. Anthroposophic physicians hold a beneficial view on fever, rarely suppress fever with antipyretics, and often use complementary means of alleviating discomfort. In AM, fever is considered to have the following potential benefits: promoting more complete recovery; preventing* infection recurrences and atopic diseases; providing a unique opportunity for caregivers to provide loving care; facilitating individual development and resilience; protecting against cancer and boosting the anticancer effects of mistletoe products.* These views are discussed with regard to the available scientific data.* Conclusion*. AM postulates that fever can be of short-term and long-term benefit in several ways; many of these opinions have become evidence-based (though still often not practiced) while others still need empirical studies to be validated, refuted, or modified.

## 1. Introduction

*Personal Note and Outline of the Structure of This Article*. We were a family with 5 children. Our parents and our family physician treated feverish illnesses with the greatest respect and always managed to make us feel safe and comfortable without suppressing the fever. None of us ever received an antipyretic (nor for that matter an antibiotic, except for the postnatal pneumonia of a prematurely born brother). The uncritical and widespread use of antipyretics and antibiotics I witnessed as a student and physician therefore seemed a strange and unphysiological interruption of a natural and, from my experience, healthy process. Our physician was an anthroposophist, so I began to enquire: What is anthroposophic medicine? What are its main statements about fever? Do these statements have any scientific basis? The first question will be addressed in Introduction; the results of the second question are presented in Results as statements. Discussion picks up each of these statements and relates them to current scientific literature.

In their 2016 editorial “Fever Phobia 35 Years Later: Did We Fail?” [1], Bertille et al. wonder why it has been so difficult to inform the world population about how to think of, and deal with, fever. They summarize “large studies seem to indicate that fever phobia persists and antipyretic drugs are still overused [2–4]. Considering that we do seem to have failed in part to provide effective guidance to parents, how did this happen?” The present review is about a cultural strain within medicine that is very successful in turning fever phobia into what could be called “fever philia”: in large hospitals [5] and thousands of practices [6] physicians and nurses interested in anthroposophic medicine have, for decades, met their patients with a contagiously appreciative attitude towards the phenomenon of fever [7, 8]. This results in a very low use of antipyretics and, perhaps more importantly, of antibiotics (5-6% in airway infections) [7–9].

*What Is Anthroposophic Medicine (AM)?* Anthroposophy is an approach to life that began its development a century ago and has inspired countless ecologic, agricultural, social, educational, economic, pharmaceutical, and therapeutic ventures throughout the world [10]. Anthroposophic physicians strive for a scientifically viable extension of their view of human nature into the realms of life and psychosocial and spiritual individuality and to perceive the interactions of these aspects and their relationship to nature, health, and illness.

In AM each illness is understood as a challenge to the human being as a whole, concerning, to various degrees, all levels of existence [11]: biological, psychosocial, and spiritual. Illnesses and instabilities are also considered to be salutogenic opportunities for developing new and sustainable balances of health, self-regulation, and development. This perspective leads to what could be called a kind of “esthetic logic,” promoting a low, and yet still safe, use of antipyretics, antibiotics, and so forth.

AM is practiced in over 80 countries. Therapeutic modalities of AM include the whole range of allopathic mainstream medicine, as well: as mineral, herbal, and animal-derived medicines, some of which are called “anthroposophic medicinal products”; counseling and pastoral care; specific therapies using movement (Bothmer Gymnastics; Spacial Dynamics, eurythmy therapy; dancing), painting and modeling (art therapy), music (music therapy), and speech (speech therapy); various forms of physiotherapy and massage (Rhythmical Massage Therapy according to Ita Wegman, Massage Therapy according to Simeon Pressel, Massage Therapy according to Volkier Bentinck, Chirophonetics, Embodiment, and others), oil dispersion baths, and external applications [12–17]. There are to date over 1500 publications on AM [6, 18]. Several hospitals, including secondary/tertiary level and regional/community hospitals, have been founded in order to broaden the spectrum of conventional medicine in this manner.

This article is about the kind of fever (body temperature > 38.5°C) that develops in response to an acute infection. The aim of this scoping review was to gather the views on fever expressed in anthroposophic literature and to assess the validity of these views with respect to the available scientific data. 

*A Note on Context.* For the past 4 years the author has, alongside his Professorship, worked as Senior Consultant in a regional hospital that has a 40-year history of thinking and dealing with fever in the way described in this paper. The author would thus classify himself as an experienced “participant observer” whose views on fever have been influenced by daily practice as well as material from lectures and articles in German, English, French, and Spanish and conversations in these languages with specialists from around the world. In the last paragraph of Section 4, the author summarizes his experience of how fever is dealt with in anthroposophic medicine.

## 2. Methods

The following searches were performed.

*Search A.* The databases PubMedCentral (PMC) and Embase were searched in February 2016 using the following terms: (anthroposophic [All Fields] OR anthroposophical [All Fields] OR anthroposophically [All Fields] OR anthroposophics [All Fields] OR anthroposophie [All Fields] OR anthroposophique [All Fields] OR anthroposophiques [All Fields] OR anthroposophisch [All Fields] OR anthroposophische [All Fields] OR anthroposophischen [All Fields] OR anthroposophischer [All Fields] OR anthroposophisches [All Fields] OR anthroposophs [All Fields] OR anthroposophy [All Fields] OR anthroposophy/history [All Fields] OR anthroposophy/psychology [All Fields]) AND (“fever” [MeSH Terms] OR “fever” [All Fields] OR “febrile” [All Fields] OR “Fieber AND anthroposoph^*∗*^” [All Fields]). 

*Search B.* CAMbase, a German bibliographical database system for complementary and alternative medicine (http://www.cambase.de/), was searched for the words “(Fieber OR Fever) and anthropos” and for “Fieber OR Fever”. Google Scholar was included to find grey literature.

Textbooks on anthroposophic medicine [19–22] were also used.

Inclusion criteria for a written publication to be used for Section 3 were as follows:

- The publication is published in a peer-reviewed journal or is a text-book on anthroposophic medicine.
- The publication contains views about fever.

There were no further exclusion criteria.

Statements that present the anthroposophic perspective on fever were extracted from the text, coded, sorted, and clustered into generic statements by the author as personal résumé and interpretation. The statements are presented in Section 3, annotated with their respective sources in anthroposophic medical literature.

*Search C.* Since the author has in the course of time collected a lot of literature in favor of these statements, he concentrated the further search on finding data that put these statements into question. The main of these searches, Search C, was performed in PubMedCentral (PMC) with the words “fever AND (detrimental or harmful)”.

## 3. Results

**Figure 1:**
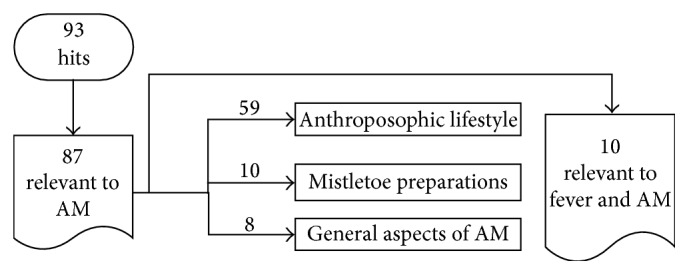
Results of literature search A on fever and anthroposophic medicine (AM).

Search A produced 93 hits from which 87 publications were relevant to fever and AM. Of these (see also Figure 1),

- 59 publications discussed or referenced either studies examining the connection between atopy and children living an “anthroposophic lifestyle” (the seminal study being the Lancet article by Alm et al. [7]), or studies where such a lifestyle impacted on low immunization levels in various communities,
- 10 publications discussed or reported the fever-inducing effect of anthroposophic cancer remedies containing* Viscum album*, some of which discussing fever as part of the anticancer action of* Viscum album *extracts,
- 8 publications discussed or referenced other or general aspects of AM not directly related to the management of fever,
- 10 publications expressed anthroposophic opinions about fever, including a focus group study of factors that influence vaccination decision-making by parents who visit an anthroposophical child welfare center [7, 8, 23–30].

The search in Embase did not add any more relevant references.

Search B did not add any more relevant references.

The findings were extracted from the text, coded, sorted, and clustered into generic statements by the author, leading to the following thirteen statements. In Section 4, each statement is discussed in the context of present scientific data:

1. The leading motif in anthroposophic medicine is that warmth and, in desease, fever are direct manifestations of the “self” working on the body, making the body more an instrument and expression of the “ego,” the “I” (in German “das Ich”) [19–22, 25–27].
2. Fever may allow faster and/or more complete resolution of infections [8, 19–23, 26–29].
3. Fever may prevent recurrent infection [19–22, 26].
4. Fever may assist immune maturation in children [19–22, 25–27].
5. Fever may protect against developing allergic diseases [7, 19–22, 26].
6. Fever may help resolve allergic disease [7, 19–22, 26].
7. Fever offers a unique opportunity for caregivers to provide loving care [19–22, 26].
8. Fever may facilitate individual development and creativity [19, 20, 22, 23, 28].
9. Febrile illness may help a developing child take ownership of their body towards a better expression of their unique individuality and to overcome inherited (e.g., epigenetic) traits [19–23, 26, 28].
10. Febrile illnesses may be protective against cancer [19–22, 24, 25, 30].
11. Some febrile illnesses may contribute to curing cancer [19, 20, 25].
12. Febrile reactions to injecting mistletoe products in cancer treatment may improve treatment outcome [19–22, 24, 30].
13. Antipyretics such as acetaminophen and ibuprofen should be used sparingly: only if other means of relieving discomfort fail or if fever needs to be suppressed for other medical reasons [8, 19–22, 25–27].

Search C, in PubMedCentral (PMC) with the words “fever AND (detrimental or harmful)”, led to 445 hits. A scan of these papers and their respective reference lists for evidence speaking against the above statements led to studies indicating that fever may also be detrimental in rare, very particular circumstances [31–38] that cooling may [39], or may not [40], be advantageous in sedated ventilated sepsis patients that measles possibly impair the immune system for 2-3 years [41]. Furthermore, fever and cytokines may interfere with fetal brain development, especially in birth-related hypoxemia, which may be the reason for naturally subdued febrile reactions in newborn (for review [42]). These studies are introduced in the respective places in Section 4.

## 4. Discussion

The discussion is structured around the anthroposophic perspectives on fever as they are stated in thirteen resulting statements listed in Section 3.

1. * The leading motif in anthroposophic medicine is that warmth and, in diesease, fever are direct manifestations of the “self” working on the body, making the body more an instrument and expression of the “ego,” the “I” (In German “das Ich”) [19–22, 25–27].*

This is a notion specific to anthroposophy. It is related to the whole breadth and depth of what human warmth is and can be, including social life and human will, and the increasingly autonomous and differentiated relationship that animals have had to warmth throughout evolution. This topic goes far beyond the scope of this article. It is, however, worth mentioning that human “warmth,” the most powerful personality trait in social judgment, is enhanced by physical warmth [43, 44].

* “Fever may allow faster and/or more complete resolution of infections” [8, 19–23, 26–29].*
Case vignette: A publication on the anthroposophic approach to pneumonia reports a 10-year-old girl with a pneumonia that developed from a flue (she had been stressed by fears of entering secondary school and much discussion about this in the family). She was treated without antibiotics and on the 5th day still had fever despite the fact that the coughs were loosening. Examination revealed cold feet and back despite high fever, upon which she was treated with 2 water bottles on her feet and massaged with a silver cream (Argentum 0.4% Ungt.) on her back. On the next day the temperature fell and 2 days later she was free of fever. The subsequent entry into secondary school was unproblematic [26].

The positive attitude of anthroposophic physicians with regard to fever has support from the evolutionary point of view. Fever is a highly conserved evolutionary host response with survival and salutary benefit. Fever is one component of the acute phase response, which is a complex physiological reaction to disease or injury, and elicits cytokine mediated rise in core temperature, generation of acute phase reactants, and activation of a number of physiologic, endocrinologic, and immunological effects [45]. Even though increasing the body temperature is energy-costly (10–12.5% increase in metabolic rate for 1°C increase in body temperature in warm-blooded animals [46]), it is an established mechanism in response to injury and infection in fish, amphibians, reptiles, birds and mammals, and many invertebrates such as insects [47]. Even cold-blooded animals seek external ways of increasing their body temperature when they are infected and their mortality increases if they are prevented from doing so [48]. Studies point to potentially harmful effects of suppressing fever in mammals and humans [16, 49–52]. The protective effects of fever against invading organisms result from a variable combination of direct thermic effects [53] and humoral [54] and cellular [55] defense enhancement. With few exceptions [53], from the point of view of immunity and survival, fever offers the host an adaptive advantage [56]. Human studies on malaria [51], chicken pox [50], and induced rhinovirus [57] infections also suggest that fever suppression delays recovery. Fever was associated with decreased mortality in Gram-negative bacteremia [58]. Hospitalized elderly patients with community-acquired pneumonia were seven times more likely to die if they did not display fever and leukocytosis [59]. There are several reports of treating Sydenham's Chorea by inducing fever [60, 61].

But if fever really were so important, would we not have genetic models of animals or humans who are unable to produce fever and have worse outcomes? There may be such models on the horizon: In a study showing that early and strong immune responses are associated with control of viral replication and recovery in Lassa virus-infected Cynomolgus monkeys, the authors noted absence of significant fever in nonsurvivors despite high levels of IL-6 [62]. In another study, sepsis patients with mitochondrial DNA haplogroup H had the best survival and the most extreme core temperature within the first 24 hours [63]. Closest to a genetic model is the report of two consanguine siblings with RANK mutations: two siblings with autosomal-recessive osteopetrosis had a markedly abrogated fever response to pneumonia and worse course of disease compared to age-matched children [64].

The improved immune function during febrile temperatures has to be weighed up against metabolic costs and potential damage to sensitive organs such as the brain, possibly the fetus [31, 32], and, in mice infected with* Klebsiella pneumoniae*, the lung [33]. Thus, in some cases, hypothermia or suppression of fever may reduce mortality, even though it increases bacterial load [34]. Nevertheless, weighing up the pros and contras leads to the conclusion that, for acute febrile infections under normal circumstances, it is better to not suppress the fever [42]. A very large recent study has shown that, even on the ICU unit, suppressing fever does not seem to convey an advantage to the patient [65].

* “Fever may prevent recurrent infection” [19–22, 26].*
* “Fever may assist immune maturation in children” [19–22, 25–27].*

In analogy to the loss of memory performance when sleep is disturbed, AM proposes that interrupting the fever phases may impair disease resolution and long-term immunity. Anthroposophic physicians report that patients, especially children, often stop having recurrent infections after experiencing an acute febrile illness without use of antipyretics or antibiotics [19–22], although studies to this regard are lacking. Conversely, there is evidence that the use of paracetamol in conjunction with vaccination can lead to less fever and reduced antibody response [66].

The findings that the risk of islet autoantibody seroconversion and subsequent development of type 1 diabetes was associated with respiratory infections during the first 6 months of life (a time at which the children are often not yet able to develop fever and when height of fever is correlated with severity of disease [67]), but not thereafter, raises interesting questions in this context [68]. On the other hand some infections, such as measles, may initially impair immunity [41].

* “Fever may protect against developing allergic diseases” [7, 19–22, 26].*
* “Fever may help resolve allergic disease” [7, 19–22, 26].*

These are topics that deserve future research. There is controversial evidence that use of acetaminophen in the first year of life and in later childhood is associated with an increased risk of asthma, rhinoconjunctivitis, and eczema in children and adults [69, 70]. However, this may be more linked to acetaminophen itself than to suppressing fever, since prenatal exposure to acetaminophen predicted wheeze at age of 5 years in an inner-city minority cohort, and the risk was modified by a functional polymorphism in GSTP1, suggesting a mechanism involving the glutathione pathway [71]. Furthermore, use of ibuprofen seems to cause less increase in asthma morbidity than use of acetaminophen [72]. Nevertheless, an inverse relationship between febrile diseases in early childhood life and allergy has been found in most studies [73–75] (one study shows a positive correlation [76] but did not control for antipyretic use), despite the fact that antipyretic [69–72] and antibiotic [75] treatment of febrile infections may increase the risk of asthma. The lower rate of atopy in younger siblings further suggests that cross-infections acquired early in infancy or childhood might prevent development of atopy [77]. Children who are raised in an anthroposophic lifestyle, which includes very restricted use of antipyretics, show less allergies [7].

* “Fever offers a unique opportunity for caregivers to provide loving care” [19–22, 26].*

Some children with autism appear to become more socially adept during and after a febrile infection [78, 79]. Such improvements are not associated with hyperthermia by high ambient temperature or exercise so alternative mechanisms mediated by acute phase cytokine actions, heat shock proteins, or alterations in the hypothalamic-pituitary-adrenal axis may be responsible for the temporary behavioral changes. Whether other children with febrile infections are particularly receptive to love and care and whether the given attention is particularly formative in this situation have not been studied to date; anecdotal evidence from those with the opportunity of caring for a febrile child or who remember their care from their own childhood seems to suggest that febrile illnesses offer a great and usually thankfully short opportunity for nurturing a relationship [28]. Studies on oxytocin levels, bonding, and empathy during febrile illnesses are lacking, while physical warmth has been shown to increase empathy, trust and generosity [43, 44]. Paracetamol, by contrast, can reduce empathy [80].

* “Fever may facilitate individual development and creativity” [19, 20, 22, 23, 28].*
* “Febrile illness may help a developing child take ownership of their body towards a better expression of their unique individuality and to overcome inherited (e.g., epigenetic) traits” [19–23, 26, 28].*

Biographical accounts of the role of acute febrile illnesses in facilitating developmental steps and helping to find new individual creativity are aspects of fever that have yet to become subject to scientific investigation. Johann W. von Goethe, for example, suffered several severe acute febrile illnesses and felt he came through each time with new impulses [81]. Laurens van der Post has written stunning words about his experience of fever and of its relationship with transpersonal past and future, for example,* “All I would suggest is that the future had begun to register a new design in my blood, and that the fever marked the beginnings of its struggle for awareness” *[82]. In anthroposophic medical practice, these considerations are part of promoting the best possible outcome. Many anthroposophic oriented caregivers, parents, and teachers believe and seem to have often experienced [19, 20, 22, 23, 26–28] that febrile illnesses offer a child the chance to optimize the process of making their body a better expression of their “true self.” It would be interesting to study how the catabolic activity of fever and the anabolic convalescence that follows it promote human development. We know that other kinds of catabolic activity, such as enthusiastic physical, musical, artistic, and mental exercise and work, do so when they are felt to be meaningful and are properly balanced by anabolic phases of nutrition, rest, and sleep. Such thoughts certainly help many parents and patients go through the process of an illness with a positive attitude and many interesting observations have been collected [19, 20, 22, 23, 26–29]. Indeed, in a study of 1001 children with measles, of whom 886 children were studied prospectively, 74% of children were judged as having profited from having measles, and 97% of parents whose children had measles said they would, in hindsight, again decide to not vaccinate their children against measles. In contrast only, 2.4% would rather have vaccinated them and 0.5% of children developed measles despite having been vaccinated [23]. However, this study may be biased by the parents potentially having a critical stance to vaccination: a recent study using population-level data suggests that measles may be followed by long-lasting immunodeficiency for about 2 years, correlating with an increase in nonmeasles related mortality during this time and perhaps explaining the disproportionately large reductions in mortality seen after the introduction of measles vaccination [41]. No such associations were found for pertussis or pertussis vaccination and the author is not aware of such studies for other acute infectious diseases. Future studies on the effects of various feverish illnesses on individual development would thus be needed to answer these questions.

* “Fever may be protective against cancer” [19–22, 24, 25, 30]. *

From an anthroposophic point of view, health arises in an actively maintained, ongoing, and dynamic balance between polarities, each of which represents a pathological direction. Living nature manifests itself everywhere within polar opposite forces, which are working together in rhythmical alternations [83]. One such polarity in living organisms is between the tendencies to form, condense, or harden on the one hand and to swell, grow, and dissolve on the other hand. The former is related to coldness and “sclerosis,” and the latter to heat and “inflammation.” From a broad developmental perspective, the young child is both psychologically and biologically closer to the warm inflammatory pole, whereas an older person is closer to the cold sclerotic pole. This may be expressed in the decrease of mean body temperature and temperature responsiveness with old age [84] and in the fact that young children are more able to develop fever and generally tolerate it better than the elderly [85]. Adults often appear to suffer more headaches and pains during fever. Some feel drained and exhausted for a long time after a feverish illness while others report feeling rejuvenated and “lighter” or “cleansed” and this may be dependent on the health of the host, the nature of the causative agent, the way the illness was dealt with, and the self-awareness of the individual (personal experience of the author; studies in this regard are lacking). This leads to the question as to whether a febrile disease can help to overcome hardening tendencies in a person's physical, immunological, and psychological make-up.

From an anthroposophic point of view, many illnesses can be assessed in terms of their relationship to the “inflammatory” and “hardening” poles: childhood cancers such as leukemias and embryonic tumors are more related to the “inflammation” pole whereas the common solid tumors of the adult develop in a more “hardening” context. Life processes that lose their organic connection with their antagonists may become isolated and move towards the direction of “hardening”: this may lead to microcalcification, autoimmune processes, or even cancer as a consequence of loss of immune surveillance. These “hardening” processes can trigger more or less helpful “inflammatory” counterregulations. While chronic inflammation can lead to sclerosis and cancer [86], there is evidence to suggest that acute febrile inflammations in children and young adults could be protective against cancer development in later life [87]. Cancer patients report a history of fewer fevers during infections than healthy controls [88, 89]. An inverse relationship between the number of children's febrile infections and the incidence of melanoma has been reported [90]. Taking these concepts into the animal world, it is interesting to note that animals with hardly any “hardening” or aging tendencies and with a strong ability to rebuild lost limbs, such as hydra, hardly ever develop cancer [91]. There is an astounding lack of in vitro and in vivo data comparing immune function and cancerogenesis at various temperatures. Recent experiments have shown that housing mice at thermoneutral temperature (30-31°C) instead of standard laboratory temperature (20–26°C) reduces tumor formation, growth rate, and metastasis. Furthermore, given the choice, tumor-bearing mice select a higher ambient temperature than non-tumor-bearing mice [92].

* Some febrile illnesses may contribute to curing cancer [19, 20, 25].*

Anthroposophic physicians may at times actually attempt to support and elicit fever, particularly in cancer treatment. Metabolic processes and biochemical reaction rates across numerous cellular functions of immune cells are increased at temperatures that simulate naturally occurring fever, for example, leucocyte proliferation, maturation, and activation, neutrophil and monocyte motility, migration, phagocytosis and pinocytosis, T cell expansions, activation and cytotoxic activity, antibody production, dendritic cell antigen processing [93] and presentation to T cells and migration to the draining lymph nodes, and lysis of bacteria and the bactericidal effect of antibiotics [94–96]. Some tumor cells may be more vulnerable to higher temperatures than healthy cells, a possibility which hyperthermia therapy of cancer attempts to address. This leads to the question as to whether the heat alone is responsible for the reported possible cancer regressions or whether actively generated fever is essential.

This question was the starting point of a fascinating story of cancer research and personal courage in the course of which surgeon Coley (re)discovered that erysipelas (streptococcal cellulitis) can lead to complete cancer remission. Of the 1200 documented patients he treated with Coley-Toxin 270 patients with nonoperable, often metastasized, cancer reportedly went into complete remission [97, 98]. In fact, about 80% of the reported spontaneous remissions from cancer are found to be related to infections [99] and the connection between febrile infection and spontaneous tumor regression is the most frequent association found in literature [86, 100–105]. This suggests that more than just increased heat is needed to fight cancer [102, 106, 107]: immunologic events that accompany some forms of fever may play a role in overcoming immunological escape mechanisms of tumor development.

* “Febrile reactions to injecting mistletoe products in cancer may improve treatment outcome” [19, 20, 20–22, 24, 30].*

The use of mistletoe (*Viscum album* L.) for cancer treatment was first suggested by Steiner, who emphasized that fever induction was essential for the success [11, 108]. After a pioneering phase with substantial (but all reversible) acute phase reaction side effects, mistletoe was mainly used in more easily tolerable doses during the past decades [109, 110]. A local inflammatory reaction at the injection site, fever and flu-like syndrome belong to the expected “adverse events” (there are several hundred publications on this [111]). The reported cases of remission, regression, and stable disease in patients treated solely with mistletoe extracts, however, suggest that high doses and more targeted use may be more likely to achieve an effect and that a strong febrile reaction to the mistletoe extract may be a prognostically positive factor [109, 112–114] and perhaps part of the anticancer effect of mistletoe [24, 112, 113, 115]. It must be noted, however, that nonfermented* Viscum album* extract usually only induces fever in the first weeks [24]. Chronothermometric examinations suggest that the fever generated by mistletoe injection may enhance endogenic rhythms, thereby increasing and harmonizing heart-rate variability [116]. Interestingly, tumor-related fever in cases of lymphomas can apparently be overcome by mistletoe-induced fever [117]. These chronothermobiological aspects of cancer therapy may become a focus of research in the future.

Mistletoe injection products have been shown to increase quality of life [18, 24, 111, 118–120] in patients with cancer and there is a wealth of literature on their immunomodulatory effects [109, 111, 121]. High-quality clinical studies on the anticancer effects of mistletoe on survival time are rare and the jury is still out as to when, how, and for whom mistletoe may be beneficial. Recent randomized trials show increased survival time in advanced pancreatic cancer [122] and osteosarcoma [123].

* “Antipyretics such as acetaminophen and ibuprofen should be used sparingly: only if other means of relieving discomfort fail or if fever needs to be suppressed for other medical reasons” [8, 19–22, 25–27].*

Although many mainstream hospitals and practices have not yet translated this into practice [124], there is broad scientific consensus that the potential benefits of the febrile reaction are to be weighed up against the discomfort or exhaustion experienced by a minority [125].

Beyond suppressing the benefits of fever, pharmacological antipyresis has its own risks: a review financed by ibuprofen distributors [126] could not convincingly disprove that ibuprofen may increase the risk of necrotising fasciitis caused by Group A Streptococcus (GAS) secondary to varicella or herpes zoster [127–130] while mice inoculated with GAS had increased wound area and mortality when receiving ibuprofen [131]. There is increasing evidence that ibuprofen in case of respiratory infections or pneumonia may facilitate empyema and complicated pneumonia in children [132–135] and adults [136], possibly via modification of neutrophil and alveolar macrophages functionality (chemotaxis, adhesion, aggregation, and degranulation [137]) and the inhibition of prostaglandin synthesis as well as via cover-up effects on subjective symptoms, thereby delaying diagnosis and treatment. This may explain the correlation between increased sales of ibuprofen for children and complicated pneumonia in France [133], although reverse causation is also possible. Further risks associated with antipyretic use include systemic reactions, asthma (especially for paracetamol [69–72]), gastrointestinal complications and anorexia [138], low white blood cell count (ibuprofen) [139], hepatic injury (paracetamol) [140], overdose (paracetamol) [140], and, extremely rarely, anaphylaxis [141, 142] (although sometimes the reaction may be due to other substances such as mannitol [143]).

The suppression of the acute phase reaction symptoms and the slightly euphorizing effect of antipyretics is likely to increase interaction with other people and the rate and duration of viral shedding, as has been shown in human volunteers [144] and ferrets [145]. Indeed, recent modeling of available data suggests a significant increase in contagion and mortality risk through antipyretics [146]. Considering further that accidental acetaminophen overdose has caused over 100 deaths per year in the USA [140], one wonders how many lives may have been saved had anthroposophic physicians popularized their positive attitude towards fever even more ([22] has been a bestseller for several years in German-speaking countries but has only recently been translated into English).

Using antipyretics to relieve symptoms may not always be straightforward: in a placebo controlled study paracetamol did not increase well-being or fluid intake but suppressed the urge to rest (which is part of an acute phase reaction seen in all mammals) [147]. Another reason often cited for treating fever is the fear of febrile seizures. However, febrile seizures tend to occur with rapidly rising temperatures in susceptible individuals and not necessarily at high temperatures [148] and are not preventable with antipyretics [149, 150]. There are no studies on the often-observed phenomenon that febrile seizures occur when the child is beginning to shiver and may thus be avoidable through rapid heat application as soon as one notices that the child may be developing fever. Febrile seizures are a terrible experience for the parents, but so-called “simple febrile seizures” are fortunately harmless and leave no neurological sequels [150, 151]. Febrile seizures must be clearly differentiated from epileptic seizures. The relationship between the latter and fever is highly variable: some are induced by fever, others are suppressed, and many are indifferent.

Anthroposophic physicians rarely resort to antipyretics but are aware, firstly, that fever can be the sign that the body is reacting against something going seriously wrong, such as meningitis or pyelonephritis. Second, just as the normal and necessary blood pressure reaction to exertion can be dangerous for some, so can fever, and there are situations in that it may be maladaptive and deleterious as reflected by naturally occurring cold-seeking behavior in such cases. A brain that has just been damaged by hypoxia may benefit from hypothermia [152]. In some extreme circumstances, such as severe sepsis in a cool environment, the cost of developing fever may exceed its benefits [34, 153, 154]. In patients without cerebral or cardiovascular problems, however, suppressing fever with antipyretics has been associated with a sevenfold increase in mortality in the Miami intensive care unit; after this study the colleagues there have become much more restrictive in their use of antipyresis ([52] and personal communication to the author). Anthroposophic physicians strive to provide patients, parents, and caregivers with information that enables them to develop views and inclinations with regard to fever that reduce “fever phobia” [155, 156] and are in harmony with the scientific facts, to offer a sense of trust and confidence in the febrile process and knowledge of when professional examination is needed [19–21, 26, 27].

## 5. Implications for Clinical Practice

Anthroposophic medicinal products are prescribed by approximately 30,000 physicians in 18 of the 27 EU member states and in 67 other countries throughout the world [157]. Caregivers should be aware of the attitudes held in anthroposophic circles and be orientated as to which attitudes stand on solid scientific grounds, which attitudes may be wrong, and which attitudes still require validation or refutation.

## 6. Summary of an “Experienced Participant Observer” on How Fever Is Dealt with in AM-Settings

Patients beginning fever due to an acute infection usually feel cold and shiver despite rising temperature. In this case they need to be warmed until they feel warm all the way into the extremities. This reduces (1) the work of increasing body temperature, (2) the uncomfortable chills, and (3) maybe even the risk of febrile seizures; there are no studies on this to date. Note that this does not apply to exceptional situations such as brain injury, in which case cooling may be advantageous. In anthroposophic households, fine wool or cotton nightwear and cotton sheets are used to maximize “breathing” capacity; cheap synthetic fibers often lead to heat congestion. Plenty of warm teas (e.g., elderflower, lime, or lemon) and, if hungry, easily digestible food (soups and fine porridges for the younger children) are recommended in this phase. Once the plateau of fever has been reached, heat can be gently released so as to prevent sweating, yet avoiding chills. The caregivers should be able to judge the warmth, breathing, color, and circulation of the patient. Cold feet need attention. Attention must also be paid to the excretory capacity of the body in terms of urine, stools, and sweat. An atmosphere of peace and quiet, free of electronic media and social stress, is seen as conductive to a good and salutogenic course of the illness. Caregivers who interpret febrile illnesses as opportunities for bonding, for cuddly times, and for stories read or told, gentle massages, songs sung, beautiful candle-light surroundings, and warm bonding can engender a lasting feeling of trust, thankfulness, and fond memories in their patients, be they young or old. Various forms of internal and external applications can be learned by parents and carers to help patients through the course of an infection. The most common applications are compresses using lemon (on feet and/or calves), ginger (on kidney/chest area), mustard (on chest), or quark (on chest), depending on the need and constitution of the patient. Enema can relieve discomfort, constipation, and dehydration (enemas must be seen critically and are only acceptable in appropriate safe settings, yet families who have learnt to deal with enemas hardly ever have to bring their small children into hospital for IV rehydration, making enemas very relevant for third-world and remote areas) [158]. Anthroposophic physicians will additionally recommend various herbal remedies depending on the symptoms, stage, and etiology of the illness [19–21, 26, 27, 159]. Two prospective outcomes studies on 12081 [9] and 1016 [7] patients with acute respiratory and ear infections suggest that anthroposophic physicians have the lowest documented antibiotic prescription rate at 6.7% [9] and 5.5% (with faster recovery, better satisfaction, and half as many side effects [7]) versus a moderate rate of 33.6% antibiotic prescription by conventional physicians in the same German study [7].

## 7. Conclusion

Scoping the available literature and views within the anthroposophic medical community led to thirteen opinion statements about fever. Many of these views are now well substantiated scientifically and slowly finding their way into the practice of mainstream medicine [124]:* Fever is a self-regulated phenomenon and does not, in a normal healthy patient with an acute infection, cause harm of itself. Going through a feverish illness may contribute to individual development and long-term health and should be accompanied in a way that fosters salutogenic competencies. Since it is self-regulated, there is no temperature above which the natural fever of acute infections must be lowered* per se in normal children [125]. Antipyretics* should be reserved for the cases in which the fever is endangering the patient, such as in severe sepsis or brain injury, where cooling is advantageous, or causing distress and malaise and alternative ways of easing their suffering fail or seem inadequate. Beware of the underlying causes of fever and carefully accompany fever instead of suppressing it*. Other views with regard to the long-term benefits of fever still await empirical confirmation, negation, or differentiation through further research.

It is important for anthroposophically inclined practitioners, carers, and patients to be aware of the fact that the scientific investigation of the effects of febrile infections on short- and long-term health and development in humans is only beginning and that many salutogenic effects [7, 8, 50, 51, 57, 59–63, 65, 67, 73–75, 77–79, 86–90, 92, 97–105] are emerging. However, fever needs special attention in late gestation [32], infancy [59] (particularly the first three months of life, babies with fever should always be promptly and thoroughly examined by a competent health worker [67]), sedated ventilated sepsis patients [39, 40], brain injury [152], and patients with Brugada syndrome (it may be recommendable to perform an ECG during fever if there is a positive family history of sudden death or syncope [37, 38], bearing in mind that 2% of the population may have asymptomatic Brugada-ECG signs in febrile conditions).
